# Sotrovimab Treatment for COVID-19 Effective in a B-Cell-Depleted Patient After Anti-CD20 Treatment

**DOI:** 10.7759/cureus.41486

**Published:** 2023-07-06

**Authors:** Kazuyuki Murase, Kohichi Takada, Yohei Arihara, Koji Miyanishi, Junji Kato

**Affiliations:** 1 Department of Medical Oncology, Sapporo Medical University, Sapporo, JPN; 2 Department of Medical Oncology, Sapporo Kiyota Hospital, Sapporo, JPN

**Keywords:** sotrovimab, hematologic malignancy, anti-cd20 treatment, b cell depletion, covid-19

## Abstract

Patients with hematologic malignancies are well known to have prolonged COVID-19 pneumonia. The cause has been reported to be B cell depletion after administration of anti-CD20 antibodies. Here, we report a case of COVID-19 pneumonia due to prolonged B-cell depletion after anti-CD20 antibody therapy for malignant lymphoma two years before. Sotrovimab, a neutralizing antibody that was designed to prevent the progression of COVID-19, was successful in preventing the progression to severe disease in this B-cell-depleted patient.

## Introduction

Severe acute respiratory syndrome virus 2 (SARS-CoV-2) causes more severe COVID-19 disease in patients with hematological malignancies than in healthy individuals [1]. If recently treated with anti-CD20 antibodies (rituximab/obinutuzumab), patients with non-Hodgkin lymphoma have impaired response to the COVID-19 vaccine and are at risk of severe COVID-19 disease. Longer time since exposure to anti-CD20 is associated with improved response rates to the COVID-19 vaccine [2]. In the lymphoma population, the recovery of the memory B-cell pool is delayed and remains below normal control levels at one year after administration of the anti-CD20 antibody rituximab [3]. Analysis of the dynamics of B-cell reconstitution after rituximab treatment shows that B-cell repopulation of the peripheral blood merely starts at six months after rituximab treatment, and more profound recovery occurs later [4]. We report a case of COVID-19 in a 63-year-old patient who had no B-cells in peripheral blood after anti-CD20 (rituximab/obinutuzumab) treatment.

## Case presentation

**Figure 1:**
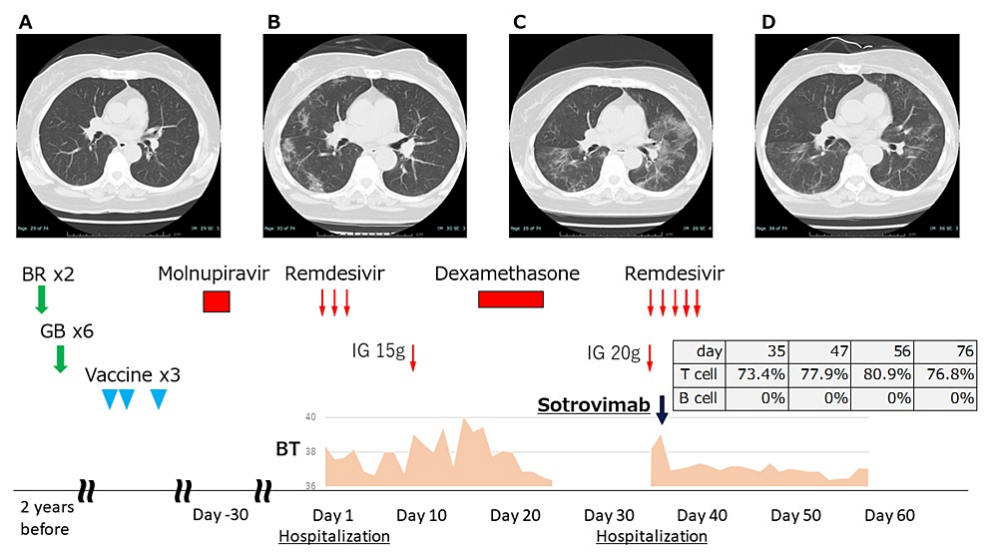
Clinical course of COVID-19 treatment with follow-up chest CT A) CT two years before; B) CT at day one; C) CT at day 34; D) CT at day 50. BR - rituximab and bendamustine; GB - obinutuzumab and bendamustine; IG - immunoglobulin; BT - body temperature °C Vaccines 13, 12, and six months before admission; molnupiravir 800 mg twice/day for five days, outpatient; remdesivir 200 mg at day one, 100 mg on days two to three; dexamethasone 6 mg/day for six days, sotrovimab 500 mg once

A 63-year-old woman was admitted to the Sapporo Medical University Hospital with a high fever (38.4°C), dry cough, and anorexia. Her medical history consisted of hypertension and obesity. Furthermore, a follicular lymphoma had been diagnosed two years before. Therapy for the follicular lymphoma consisted of two courses of rituximab and bendamustine and four courses of obinutuzumab and bendamustine during a six-month period (Figure 1). The change to obinutuzumab was because of a rash that appeared as a side effect of rituximab. After a PET-CT showed complete remission, she was followed in the outpatient clinic. She had contracted COVID-19 a month before hospitalization despite having received three doses of the BNT162b2 mRNA COVID-19 vaccine (Pfizer®) at 13, 12, and six months, respectively, before her first hospitalization. At that time, she was prescribed molnupiravir at another hospital and stayed at home. Her symptoms briefly improved but subsequently worsened, prompting her visit to our hospital. She had experienced fever, cough, and anorexia for the previous 11 days and worsening of these symptoms on the day of admission. The laboratory showed leukopenia (2600 /µL) with remarkable lymphopenia (338 /µL) and hypogammaglobulinemia (582 mg/dL). The nasopharyngeal SARS-CoV-2 RT-PCR (Cobas®) and antigen (FUJIREBIO®) results were positive. A CT scan on day one (Figure 1B) showed bilateral consolidations and ground-glass opacifications consistent with a diagnosis of COVID-19-associated pneumonia. A three-day course of remdesivir (200 mg on day one, 100 mg on days two to three) was administered. Cultures of sputum and blood were negative for the growth of bacteria or fungi. High fever over 38°C persisted for 11 days after admission. We administered 15 g of immunoglobulin, continued intravenous nutrition, and symptomatic treatment on day 12. She did not require oxygen administration but had a persistent high fever, fatigue, and anorexia. We consulted with our infection control department and started dexamethasone (6 mg once daily) for symptomatic improvement on day 17 and continued it until day 22. The patient progressively recovered and was discharged on day 24. However, her symptoms quickly worsened, and she was readmitted on day 34. Her symptoms were also high fever, fatigue, and anorexia, but she did not require oxygen administration during this second admission. CT scan (Figure 1C) showed worsening pneumonia, and we administered remdesivir and 20 g of immunoglobulin on day 34. The laboratory showed leukopenia (2900 /µL) with severe lymphopenia (145 /µL). At this time, B-lymphocyte count and serum S-antibody titer were checked. B-cell count was zero, and COVID-19 antibodies were negative. After consulting with the infection control department, we decided that neutralizing antibodies would be effective in patients who did not have antibodies. Therefore, we immediately administered the neutralizing antibody sotrovimab (500 mg once) on day 35. The next day her fever subsided, and her other symptoms improved. We followed the patient thereafter, but no recurrence of symptoms was observed. Lymphocyte count recovered from 304 to 540 /µL; however, B-cell count was also zero on days 47 and 54. Finally, her performance status improved after rehabilitation, CT scan (Figure 1D) showed improving pneumonia on day 50, and she was discharged on day 56.

## Discussion

COVID-19 has spread worldwide. While mRNA COVID-19 vaccination is effective in preventing COVID-19 [5], patients treated with anti-CD20 are unable to mount a meaningful immune response to the vaccine [6-9]. In this study, the patient received three doses of COVID-19 vaccine and was infected with COVID-19 before admission. Usually, serum antibody titer peaks at about 20 days after the onset of symptoms and gradually decreases for several months thereafter [10]. Nevertheless, her COVID-19 antibody titer on day 34 was negative due to B cell depletion. Previous reports show that a few CD19+ B cells are detectable in peripheral blood five months to one year after the end of treatment [3, 4, 11]. However, her B cell depletion was prolonged for two years. It is known that COVID-19 infection is more severe and prolonged in an immunocompromised host [12, 13], and COVID-19 patients with hematological malignancies have a twofold increased risk of death [14]. Molnupiravir targets the viral polymerase, causes lethal mutations within the virus during replication, and decreases severe outcomes [15]. Remdesivir, an inhibitor of the viral RNA-dependent RNA polymerase with in vitro inhibitory activity against SARS-CoV-1, is superior to placebo in shortening the time to recovery [16]. Dexamethasone also has been reported to be effective in COVID-19 treatment [17]. In this report, she was prescribed molnupiravir, and we added remdesivir twice and dexamethasone therapy. However, these treatments did not lead to a complete cure for COVID-19. Therefore, we speculated that patients who did not have COVID-19 antibodies due to B cell depletion would benefit from neutralizing antibodies. Sotrovimab is a neutralizing antibody designed to prevent the progression of COVID-19 [18, 19]. A similar case report also showed that sotrovimab led to rapid improvement in two patients with persistent COVID-19 after B cell depletion [20]. After administration of sotrovimab, her fever quickly resolved, and her symptoms were not further exacerbated. A re-examination of the patient one month after hospital discharge showed that B cells remained depleted, but there was no relapse of COVID-19 pneumonia.

## Conclusions

B cell depletion after anti-CD20 antibody therapy for malignant lymphoma causes more severe COVID-19 disease. Sometimes patients have persistent B cell depletion more than two years after anti-CD20 antibody treatment, as reported here. Therefore, it is useful to check antibody titers and B cell ratios in cases of refractory COVID-19 infection. In addition, the results suggest that neutralizing antibodies may be effective in patients with B cell depletion and without COVID-19 antibodies, as in this case. Prospective controlled cohort studies would be beneficial to validate the findings and strengthen the conclusions of this report.
